# Therapeutic Exosomes in Prognosis and Developments of Coronary Artery Disease

**DOI:** 10.3389/fcvm.2021.691548

**Published:** 2021-05-31

**Authors:** Ai-Qun Chen, Xiao-Fei Gao, Zhi-Mei Wang, Feng Wang, Shuai Luo, Yue Gu, Jun-Jie Zhang, Shao-Liang Chen

**Affiliations:** ^1^Department of Cardiology, Nanjing First Hospital, Nanjing Medical University, Nanjing, China; ^2^Department of Cardiology, Nanjing Heart Centre, Nanjing, China

**Keywords:** exosomes, CAD, atherosclerosis, myocardial infarction, drug delivery

## Abstract

Exosomes, with an diameter of 30~150 nm, could be released from almost all types of cells, which contain diverse effective constituent, such as RNAs, proteins, lipids, and so on. In recent years, exosomes have been verified to play an important role in mechanism, diagnosis, treatment, and prognosis of cardiovascular disease, especially coronary artery disease (CAD). Moreover, it has also been shown that exosomes derived from different cell types have various biological functions based on the cell stimulation and microenvironment. However, therapeutic exosomes are currently far away from clinical translation, despite it is full of hope. In this review, we summarize an update of the recent studies and systematic knowledge of therapeutic exosomes in atherosclerosis, myocardial infarction, and in-stent restenosis, which might provide a novel insight into the treatment of CAD and promote the potential clinical application of therapeutic exosomes.

## Introduction

Coronary artery disease (CAD) still remains a high-prevalence, high-risk, and high-fatality cardiovascular disease worldwide. In spite of the profound development of device and agents in CAD treatment, the prognosis of CAD, especially acute myocardial infarction, is far from being satisfactory (1, 2). Recently, exosome emerges as a novel, full of hope, and potential alternative to cell-based therapies of CAD due to its cardioprotective properties (3).

**Figure 1:**
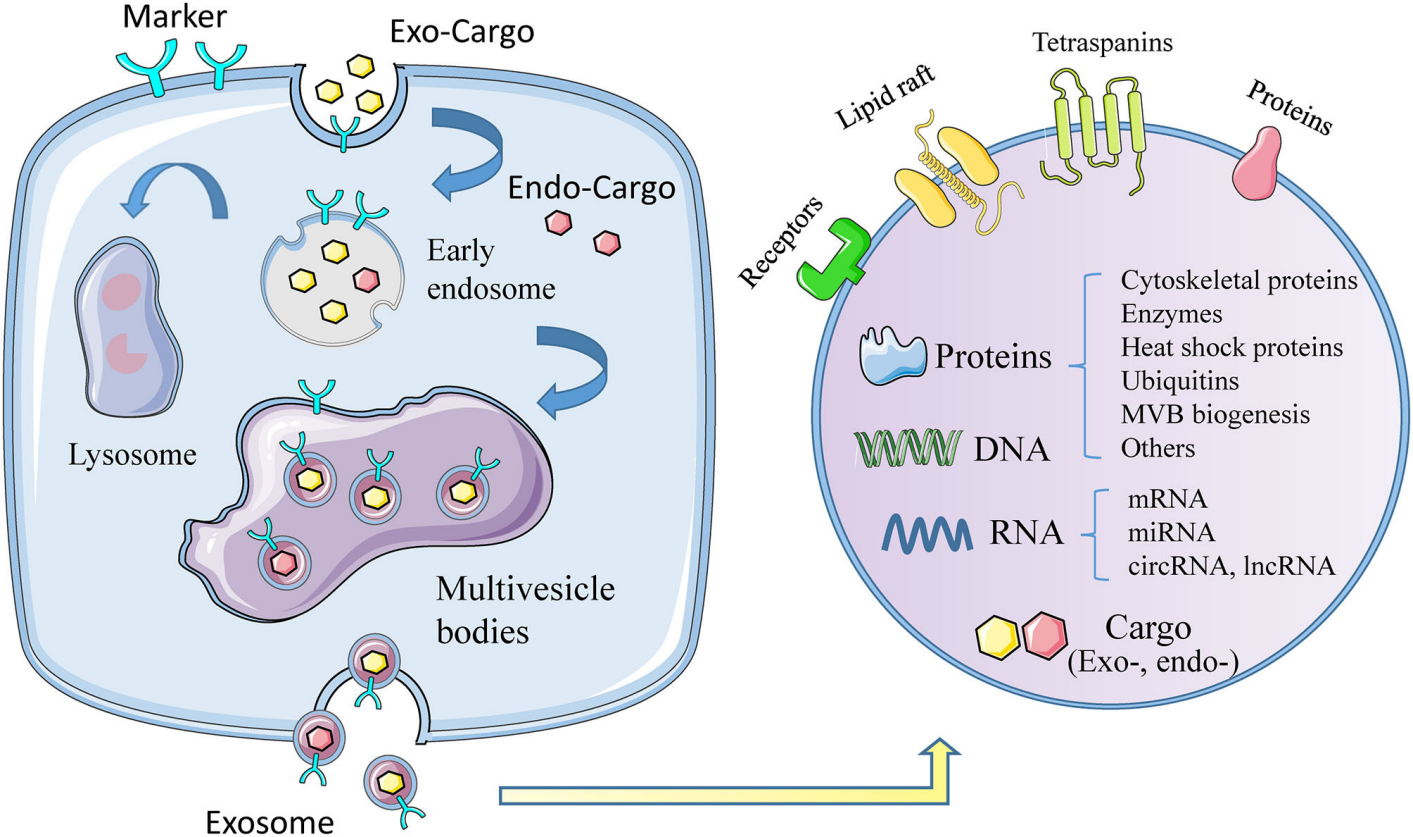
The biogenesis, formation, and content of exosomes. The formation of exosomes begins with invagination of the plasma membrane, and then forms early exosomes, which form multiple vesicles after fusion, and finally release the exosomes out of the cell. During the formation of exosomes, it will actively or passively carry exogenous or endogenous cargoes. The picture on the right shows the membrane structure of exosomes, the contents of exosomes, including proteins, DNA, RNA, and others.

Exosomes, with diameter of 30~150 nm and density of 1.13~1.19 g/ml, are the smallest extracellular vesicles (EVs) (4), with a bilayer membrane structure released by almost all types of cells (5, 6). The biogenesis of exosomes triggers from membrane proteins being endocytosed via inward budding of the cell membrane, which are then transferred to early endosomes (EEs). Afterwards, the EEs mature into multivesicle bodies (MVBs), filled with numerous intraluminal vesicles (ILVs) (7, 8), which incorporate proteins, lipids, and genetic material during invagination (9). Finally, MVBs can fuse with cell membrane and release ILVs to the extracellular space (10), as we call them exosomes, or result in degradation via fusing with lysosomes (Figure 1) (11).

However, therapeutic exosomes are currently far away from clinical application, in spite of so many outstanding qualities of exosomes. In this review, we will summarize an update of the recent findings and systematic knowledge of therapeutic exosomes in CAD, which might provide a novel insight into the treatment of CAD and promote the potential clinical translation of therapeutic exosomes.

## Exosomes and CAD

According to the progress of CAD, the relationships between exosomes and CAD are summarized into three parts: exosomes in the prevention of atherosclerosis, exosomes in the diagnosis and treatment of myocardial infarction, and exosomes in the development of in-stent restenosis (Table 1).

**Table 1:** Relationship between exosomes and CAD.

**Disease**	**Exosomal cargo**	**Parent cells**	**Recipient cells**	**Target**	**Biological/clinical relevance**	**Reference**
AS	miR-223	THP-1 monocyte	HUVEC	STAT-3 pathway	Anti-inflammation	(12)
	HSP27	THP-1 monocyte	–	NF-κB, IL-10	Anti-inflammation	(13)
	Mitochondria	Monocyte	Endothelial cell	IFN, TNF	Anti-inflammation	(14)
	miR-1	Hepatocyte	Endothelial cell	KLF4, NF-κB	Anti-inflammation	(15)
	miR-21-3p	MACROPHAGE	VSMC	PTEN	Promote VSMC proliferation and degradation	(16)
	–	Gastric epithelial cell	Macrophage	CagA	Promote foam cell formation	(17)
	Sonic hedgehog	Adipocyte	HUVECs, MAECs	TGF-α, IL-1β, IL-6	Reduce plaque vulnerability	(18)
MI	miR-342-5p	Endothelials	CMs	Caspase9, Jnk2, Akt	Anti-apoptsis/proliferation	(19)
	miR-21	HEK293T cell	CMs, HUVECs	PDCD4	Anti-apoptosis	(20)
	miR-125b-5p	MSC	CMs	p53, BAK1	Anti-apoptosis	(21)
	miR-210	EPC	Endothelial cell	Mitochondria	Anti-apoptosis/promote angiogenic function	(22)
	miR-24	Serum	H9c2 cell	Bim	Mediate Remote ischemic preconditioning	(23)
	miR-93-5p	Adipose stromal cell	CMs	Atg7, TLR4	Inhibit autophagy, anti-inflammatory	(24)
	lncR	–	Fibroblast, CMs	Neat1	Anti-fibrosis	(25)
	miR-24	MSC	CD8+T	Bim	Anti-fibrosis	(26)
	miR-130-3p	Adipocyte	CMs	AMPKα1/α2, Birc6, and Ucp3	Anti-apoptosis (diabetic)	
	Cytotoxic substance	Serum	HL-1 CMs	Compliment C4, ApoE, Apo C-IV	Anti-apoptosis (diabetic)	(27)
	ILK	Progenitor	CMs	NF-κB	Enhance myocardial repair	(28)
ISR	miR-222	M1-macrophages	VSMC	CDKN1B/CDKN1C	Promote VSMC proliferation and degradation	(29)
	miR-125b	MSC	VSMC	Myosin-1E	Promote VSMC proliferation and degradation	(30)
	miR-21-5p	EPC	HUVEC	THBS1	Promote repair of endothelial cells	(31)

*AS, atherosclerosis; MI, myocardial infraction; ISR, in stent restenosis; MSC, mesenchymal stem cell; EPC, endothelial progenitor cell; CM, cardiomyocyte; VSMC, vascular smooth muscle cell; HUVEC, human umbilical vein endothelial cell*.

### Therapeutic Exosomes in Atherosclerosis

A basic progress in the development of atherosclerosis is monocytes/macrophages accumulation into the vessel wall to produce pro-inflammatory cytokines (32). It has been reported that molecularly engineered M2 macrophage-derived exosomes (Further electroporated with hexyl 5-aminolevulinate hydrochloride) alleviated inflammation by promoting the release of anti-inflammatory cytokines (33). Paeonol could restrict atherosclerosis by obviously increasing miR-223 expression in exosomes from monocytes and inhibiting STAT3 pathway (34). Exosomes laden with heat shock protein 27 (HSP27) significantly stimulated NF-κB activation and IL-10 release, suggesting that exosomes could act as a vector in anti-inflammatory therapy (35). Mitochondria constituted a major subset of extracellular vesicles released by LPS-activated monocytes *in vitro*, which were associated with type I IFN and TNF signaling (36). Exosomes from nicotine-stimulated macrophages could promote atherosclerosis through facilitating VSMC migration and proliferation by targeting miR-21-3p/*PTEN* (37). Moreover, helicobacter pylori-infected gastric epithelial cells-derived exosomes accelerated macrophage foam cell formation and promoted atherosclerosis by CagA (38). Insulin resistance adipocyte-derived exosomes (IRADEs) has been reported to aggravate the plaque burden, whereas its effect could be attenuated by silencing sonic hedgehog in IRADEs (12). Besides, Jiang et al. (13) also reported that steatotic hepatocyte-derived EVs promoted endothelial inflammation by miR-1 delivery, KLF4 suppression and the NF-κB pathway activation. And in this instance, exosome therapy might be the reduction of negative contents in exosomes such as miR-1 instead of increasing therapeutic exosomes.

### Therapeutic Exosomes in Myocardial Infarction

Myocardial infarction, which often results in poor clinical outcomes, still remains the lack of effective treatment, especially for those without culprit vessel revascularization (14). Therefore, current clinical treatments are mostly based on easinesss of symptoms rather than repairing infarcted cardiomyocyte (15).

Exosomes reveal significant anti-apoptosis of cardiomyocyte after myocardial infarction. Exercise-derived exosomal miR-342-5p inhibited cardiomyocyte apoptosis by targeting *Caspase9* and *Jnk2* after left anterior descending artery occlusion (16). EVs overexpressing miR-21 could dramatically reduce PDCD4 expression and alleviate myocardial apoptosis (15). Hypoxia-conditioned bone marrow-mesenchymal stem cells (MSCs)-derived exosomes (Hypo-Exo) could also protect cardiomyocytes from apoptosis by enrichment of miR-125b-5p and suppressing the expression of genes *p53* and *BAK1* (17). In addition, miR-210 in endothelial progenitor cell-derived exosomes (EPC-EXs) possessed antiapoptotic functions onto hypoxia/reoxygenation-injured human endothelial cells (18). Remote ischemic preconditioning-induced exosomes (RIPC-Exo) also could transfer miR-24 into myocardium to inhibit apoptosis (39).

Exosomes also provide cardioprotection by activating cell survival signals, inhibiting inflammatory factors, delaying ventricular remodeling, and reducing myocardial fibrosis after the occurrence of myocardial infarction. Exercise-derived exosome (Ex-exo) could carry miR-342-5p to promote Akt phosphorylation by targeting gene *Ppmlf* (16). MiR-93-5p in adipose stromal cell-derived exosomes (ADSC-Exo) inhibited inflammatory response and prevented myocardial infarction by targeting *Atg7* and *TLR4* (20). Kenneweg et al. (19) had reported that fibroblasts absorbed lncR-EVs and promoted myocardial fibrosis by targeting *Neat1*. Moreover, exosomal miR-24, derived from allogenic human umbilical MSC, could inhibit cardiac fibrosis (21).

Patients suffering from myocardial infarction often have a history of diabetes. Gan et al. (22) had demonstrated that the enrichment of miR-130b-3p from dysfunctional adipocyte exacerbated myocardial infarction and cardiomyocyte apoptosis. Serum-exosomes from normoglycemic rats could alleviate the death of hypoxia/reoxygenation-induced *HL-1* cell, however, which disappears in type-2 diabetes rat model (23).

Exosomes also can serve as an adjuvant therapy. Integrin Linked Kinase (ILK) acted as a target kinase by which progenitor cell-derived exosomes attenuated myocardial injury (24). Cheng et al. (25) have reported that miRNA in EVs contributed to early detection of CAD by means of point-of care applications.

### Therapeutic Exosomes in In-stent Restenosis

Percutaneous coronary intervention has become a very important treatment strategy for CAD, but in-stent restenosis is blamed for the main cause of stent failure in patients with CAD (26, 40). Several previous studies have shown that the risk of in-stent restenosis in CAD patients undergoing coronary stent implantation during 1 year follow-up was ~5–10% (27). The underlying mechanisms of in-stent restenosis are quite complex, and at least exosomes play a crucial role in the development of in-stent restenosis. For example, miR-222 from M1 macrophages (M1M)-derived exosomes promoted vascular smooth muscle cells (VSMCs) proliferation and migration, which resulted in restenosis (41). Wang et al. (42) reported that MSC-Exo enriched miR-125b and inhibited the proliferation and migration of VSMC by targeting myosin 1E. Moreover, EPC-Exo also were involved in the prevention of restenosis through delivering miR-21-5p and inhibiting *THBS1* expression (43). Recently, exosome-eluting stents have been proven to reduce intimal hyperplasia and accelerate re-endothelialization in the ischemic injury rat model.

## Optimized Treatment Strategy

**Figure 2:**
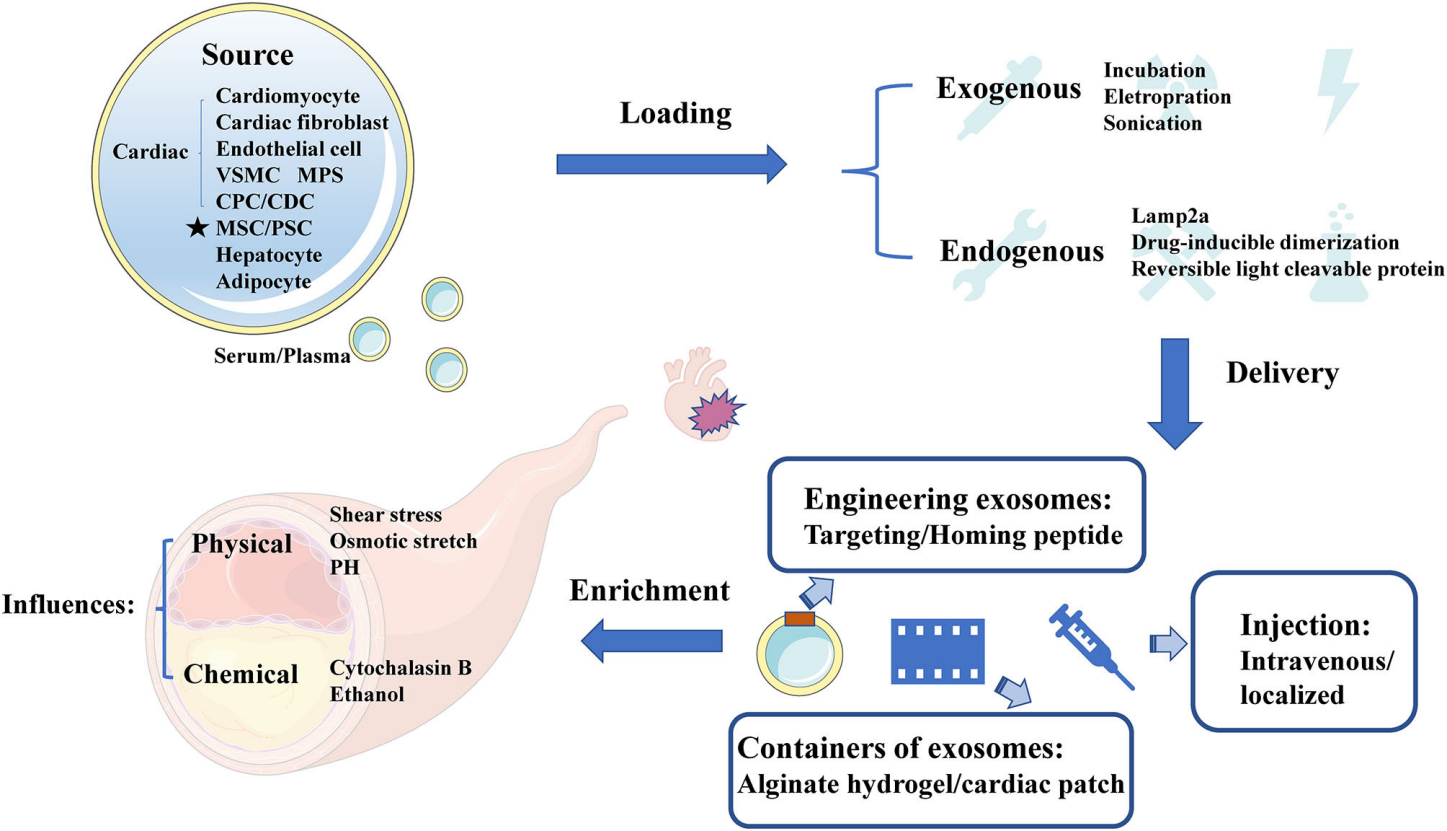
The sources, cargo loading, delivery, and enrichment of therapeutic exosomes. Therapeutic exosomes originate from a variety of cells, including some derived from cardiovascular cells, stem cells, and others. Next, we introduce the method of carrying endogenous or exogenous goods. It then summarizes the optimization strategies for exosome delivery, including targeting peptides, novel exosome containers, and injection methods for exosomes. Finally, we analyze the influencing factors of the enrichment efficiency of exosomes. VSMC, vascular smooth muscle cell; MPS, mononuclear phagocyte system; CPC, cardiac progenitor cell; CDC, cardiosphere-derived cells; MSC, mesenchymal stem cell; PSC, pluripotent stem cell; Cltc, clathrin heavy chain.

Exosomes appear superiority and irreplaceable biological functions, and the clinical application of therapeutic exosomes is full of hope. In the first place, exosomes can avoid phagocytosis and bypass the engulfment by lysosomes (44) to exhibit a longer circulation half-life due to the protection of phospholipid bilayer membrane (28). Secondly, phospholipid bilayer of exosomes is also beneficial to the fusion with membrane of recipient cells (29). Thirdly, exosomes derived from animals or patients have the high homolog and low immune response to avoid exosomes degradation (30). Finally, exosomal regulation of “Homing” effect has been reported to target the cell type where exosomes were produced (31), which can provide a shortcut for exosomes delivery. In need of optimized treatment strategy, we summarized the latest research involved of sources, cargo loading, delivery and enrichment of therapeutic exosomes (Figure 2).

### Source of Therapeutic Exosomes

It has been reported that the sources of CAD related therapeutic exosomes were commonly cardiovascular-derived endothelial cells, smooth muscle cells, macrophages and cardiac fibroblasts (45). In recent years, several studies have highlighted the value of MSC-Exo therapy in cardiac protection (46, 47), and MSC could secret the highest amount of exosomes (48). Moreover, other studies found that circulating-Exo, adipocyte-EVs (12), hepatocyte-EVs (13), accompanied with different degrees of heterogeneity, all existed therapeutically effect upon CAD.

### Loading Therapeutic Cargo in Exosomes

Although many therapeutic cargoes are inherent in parent cells previously, some therapeutic cargoes could only be loaded into exosomes by artificial means. Normally, cargoes could be loaded through fusion with liposomes, adsorption of molecules to the surface of exosomes and the insertion of lipids (49). It has been reported that a few procedures, such as incubation, electroporation (33), sonication (50), and so on (51), could promote cargo loading. When choosing the loading method of cargoes, we should consider the loading efficiency (52), and whether this loading method will change the physical and chemical characteristics of exosomes (53). Besides, membrane protein Lamp2a could increase the loading of miRNA into EVs (54). Moreover, drug-inducible dimerization (55), reversible light cleavable protein (56), and several advanced means of engineering exosomes also contribute to the loading of endogenous cargoes.

### Delivery Method

Normally, therapeutic exosomes were injected intravenously and act on the cardiovascular diseases through the circulatory system as an essential treatment. However, most of these exosomes are taken up by liver or spleen (57). Loading homing peptides has become a popular way to optimize delivery of exosomes (58). In cardiovascular field, several homing peptides in connection with atherosclerosis (59, 60), and ischemia/reperfusion–injured cardiomyocytes (61) have been identified and applied in therapeutic regimen. For example, Wang et al. (62) have demonstrated that engineered exosomes fused with ischemic myocardium-targeting peptide (IMTP) increasingly accumulated in ischemic heart area. Furthermore, it has been reported that exosomes conjugated with cardiac homing peptide (CHP) has higher retention in infarcted heart (63).

Besides, Song et al. (15) have reported that localized injection of EVs attenuated the apoptosis of cardiomyocytes and endothelial cells in a preclinical myocardial infarction (MI) animal model. To reduce losses during transportation, Lv et al. (64) have reported that sEVs, incorporated in alginate hydrogel, act as a new regimen of therapy. An off-the-shelf therapeutic cardiac patch, composed of extracellular matrix and cardiac stromal cells (CSC), has been confirmed in the model of MI (65). The examples above demonstrate the superiority of local delivery of exosomes and improve the retention rate of exosomes.

### Enrichment Efficiency

The enrichment efficiency of exosomes is affected by physical and chemical stimuli. The physical stimulation of exosomes mainly includes shear stress, osmotic stretch, PH and others (66). More importantly, the change of blood flow shear force, as the initiating factor of coronary artery disease, has also become a difficult problem for exosome delivery. Here, we focus on the shear stress in vessel where exosomes were regulated. While shear stress remain within 1–70 dynes/cm^2^ in normal blood vessels, severely narrowed blood vessels can produce over 1,000 dynes/cm^2^ (67). High shear stress, occurring in atherosclerotic arteries, could accelerate the release of circulating-EVs gradually (68). The mechanisms of shear stress on EVs secretion relate to the response of membrane tension (69). Besides, calcium could enhance exosomes secretion from a microenvironment perspective (70), whereas arterial hypertension was also associated with the increase of shear stress from a macro perspective (71). Evidence proved that exercise training could increase EVs release under high shear stress, and decrease the risk of thrombosis correspond to stenotic arteries (72). Exosomes could also be affected by chemical trigger, including cytochalasin B and ethanol (46).

## Conclusion and Future Perspective

In recent years, the therapeutic effect of exosomes on heart diseases has been gradually discovered. We have summarized the progress in studying exosomes as drug delivery vehicles. Before entering the clinical transformation, a perfect therapeutic concept of exosomes is essential (3), and pioneering in the field of exosomes is tumor-related studies. We can draw on tumor-related studies to optimize treatment regimens. Certainly, CAD-targeted treatment options also need to take notice of the cardiovascular lineage specificity.

Exosomes, as natural drug delivery vehicles, have excellent biocompatibility and targeting properties. We have discovered the potential of exosomes in the treatment of CAD based on existing research. However, exosomes still face huge resistance in clinical transformation. Moreover, we hope that the optimization of therapeutic exosomes is getting better and enter the clinical application stage as soon as possible.

## Author Contributions

A-QC and X-FG wrote the manuscript. Z-MW and FW prepared the figures. SL and YG prepared the table. J-JZ and S-LC provided the idea and revised the manuscript. All authors have agreed to the published version of the manuscript.

## Conflict of Interest

The authors declare that the research was conducted in the absence of any commercial or financial relationships that could be construed as a potential conflict of interest.

## References

[1] Sousa-Uva M Neumann FJ Ahlsson A Alfonso F Banning AP Benedetto U. 2018 ESC/EACTS Guidelines on myocardial revascularization. Eur J Cardio Thorac Surg. (2019) 55:4–90. 10.1093/ejcts/ezy289 30165632

[2] Benjamin EJ Muntner P Alonso A Bittencourt MS Callaway CW Carson AP. Heart disease and stroke statistics-2019 update: a report from the American Heart Association. Circulation. (2019) 139:e56–e528. 10.1161/CIR.0000000000000659 30700139

[3] Nazari-Shafti TZ Stamm C Falk V Emmert MY. Exosomes for cardioprotection: are we ready for clinical translation? Eur Heart J. (2019). 40:953–6. 10.1093/eurheartj/ehz106 30896768

[4] Shao H Im H Castro CM Breakefield X Weissleder R Lee H. New technologies for analysis of extracellular vesicles. Chem Rev. (2018) 118:1917–50. 10.1021/acs.chemrev.7b00534 29384376 PMC6029891

[5] Théry C Zitvogel L Amigorena S. Exosomes: composition, biogenesis and function. Nat Rev Immunol. (2002) 2:569–79. 10.1038/nri855 12154376

[6] Kalluri R LeBleu VS. The biology, function, and biomedical applications of exosomes. Science. (2020) 367:eaau6977. 10.1126/science.aau6977 32029601 PMC7717626

[7] Février B Raposo G. Exosomes: endosomal-derived vesicles shipping extracellular messages. Curr Opin Cell Biol. (2004) 16:415–21. 10.1016/j.ceb.2004.06.003 15261674

[8] Merchant ML Rood IM Deegens JKJ Klein JB. Isolation and characterization of urinary extracellular vesicles: implications for biomarker discovery. Nat Rev Nephrol. (2017) 13:731–49. 10.1038/nrneph.2017.148 29081510 PMC5941934

[9] van Niel G D'Angelo G Raposo G. Shedding light on the cell biology of extracellular vesicles. Nat Rev Mol Cell Biol. (2018) 19:213–28. 10.1038/nrm.2017.125 29339798

[10] Lo Cicero A Stahl PD Raposo G. Extracellular vesicles shuffling intercellular messages: for good or for bad. Curr Opin Cell Biol. (2015). 35:69–77. 10.1016/j.ceb.2015.04.013 26001269

[11] Raiborg C Stenmark H. The ESCRT machinery in endosomal sorting of ubiquitylated membrane proteins. Nature. (2009) 458:445–52. 10.1038/nature07961 19325624

[12] Wang F Chen FF Shang YY Li Y Wang ZH Han L. Insulin resistance adipocyte-derived exosomes aggravate atherosclerosis by increasing vasa vasorum angiogenesis in diabetic ApoE(-/-) mice. Int J Cardiol. (2018) 265:181–7. 10.1016/j.ijcard.2018.04.028 29685689

[13] Jiang F Chen Q Wang W Ling Y Yan Y Xia P. Hepatocyte-derived extracellular vesicles promote endothelial inflammation and atherogenesis via microRNA-1. J Hepatol. (2020) 72:156–66. 10.1016/j.jhep.2019.09.014 31568800

[14] Rao SV Kaul P Newby LK Lincoff AM Hochman J Harrington RA. Poverty, process of care, and outcome in acute coronary syndromes. J Am Coll Cardiol. (2003) 41:1948–54. 10.1016/S0735-1097(03)00402-9 12798563

[15] Song Y Zhang C Zhang J Jiao Z Dong N Wang G. Localized injection of miRNA-21-enriched extracellular vesicles effectively restores cardiac function after myocardial infarction. Theranostics. (2019) 9:2346–60. 10.7150/thno.29945 31149048 PMC6531307

[16] Hou Z Qin X Hu Y Zhang X Li G Wu J. Longterm exercise-derived exosomal miR-342-5p: a novel exerkine for cardioprotection. Circ Res. (2019) 124:1386–400. 10.1161/CIRCRESAHA.118.314635 30879399

[17] Zhu LP Tian T Wang JY He JN Chen T Pan M. Hypoxia-elicited mesenchymal stem cell-derived exosomes facilitates cardiac repair through miR-125b-mediated prevention of cell death in myocardial infarction. Theranostics. (2018) 8:6163–77. 10.7150/thno.28021 30613290 PMC6299684

[18] Ma X Wang J Li J Ma C Chen S Lei W. Loading MiR-210 in endothelial progenitor cells derived exosomes boosts their beneficial effects on hypoxia/reoxygeneation-injured human endothelial cells via protecting mitochondrial function. Cell Physiol Biochem. (2018) 46:664–75. 10.1159/000488635 29621777

[19] Kenneweg F Bang C Xiao K Boulanger CM Loyer X Mazlan S. Long noncoding RNA-enriched vesicles secreted by hypoxic cardiomyocytes drive cardiac fibrosis. Mol Ther Nucleic Acids. (2019) 18:363–74. 10.1016/j.omtn.2019.09.003 31634682 PMC6807307

[20] Liu J Jiang M Deng S Lu J Huang H Zhang Y. miR-93-5p-containing exosomes treatment attenuates acute myocardial infarction-induced myocardial damage. Mol Ther Nucleic Acids. (2018) 11:103–15. 10.1016/j.omtn.2018.01.010 29858047 PMC5852413

[21] Shao L Zhang Y Pan X Liu B Liang C Zhang Y. Knockout of β-2 microglobulin enhances cardiac repair by modulating exosome imprinting and inhibiting stem cell-induced immune rejection. Cell Mol Life Sci. (2020) 77:937–52. 10.1007/s00018-019-03220-3 31312880 PMC11104803

[22] Gan L Xie D Liu J Lau WB Christopher TA Lopez B. Small extracellular microvesicles mediated pathological communications between dysfunctional adipocytes and cardiomyocytes as a novel mechanisms exacerbating ischemia/reperfusion injury in diabetic mice. Circulation. (2020) 141:968–83. 10.1161/CIRCULATIONAHA.119.042640 31918577 PMC7093230

[23] Wider J Undyala VVR Whittaker P Woods J Chen X Przyklenk K. Remote ischemic preconditioning fails to reduce infarct size in the Zucker fatty rat model of type-2 diabetes: role of defective humoral communication. Basic Res Cardiol. (2018) 113:16. 10.1007/s00395-018-0674-1 29524006 PMC6776086

[24] Yue Y Wang C Benedict C Huang G Truongcao M Roy R. Interleukin-10 deficiency alters endothelial progenitor cell-derived exosome reparative effect on myocardial repair via integrin-linked kinase enrichment. Circ Res. (2020) 126:315–29. 10.1161/CIRCRESAHA.119.315829 31815595 PMC7015105

[25] Cheng HL Fu CY Kuo WC Chen YW Chen YS Lee YM. Detecting miRNA biomarkers from extracellular vesicles for cardiovascular disease with a microfluidic system. Lab Chip. (2018) 18:2917–25. 10.1039/C8LC00386F 30118128

[26] Zhang J Gao X Kan J Ge Z Han L Lu S. Intravascular ultrasound versus angiography-guided drug-eluting stent implantation: the ULTIMATE trial. J Am Coll Cardiol. (2018) 72:3126–37. 10.1016/j.jacc.2018.09.013 30261237

[27] Gao XF Kan J Zhang YJ Zhang JJ Tian NL Ye F. Comparison of one-year clinical outcomes between intravascular ultrasound-guided versus angiography-guided implantation of drug-eluting stents for left main lesions: a single-center analysis of a 1, 016-patient cohort. Patient Prefer Adherence. (2014) 8:1299–309. 10.2147/PPA.S65768 25278749 PMC4179827

[28] Saunderson SC Dunn AC Crocker PR McLellan AD. CD169 mediates the capture of exosomes in spleen and lymph node. Blood. (2014) 123:208–16. 10.1182/blood-2013-03-489732 24255917 PMC3888287

[29] Mathivanan S Ji H Simpson RJ. Exosomes: extracellular organelles important in intercellular communication. J Proteomics. (2010) 73:1907–20. 10.1016/j.jprot.2010.06.006 20601276

[30] Ha D Yang N Nadithe V. Exosomes as therapeutic drug carriers and delivery vehicles across biological membranes: current perspectives and future challenges. Acta Pharm Sin B. (2016) 6:287–96. 10.1016/j.apsb.2016.02.001 27471669 PMC4951582

[31] Park EJ Prajuabjinda O Soe ZY Darkwah S Appiah MG Kawamoto E. Exosomal regulation of lymphocyte homing to the gut. Blood Adv. (2019) 3:1–11. 10.1182/bloodadvances.2018024877 30591532 PMC6325302

[32] Libby P Okamoto Y Rocha VZ Folco E. Inflammation in atherosclerosis: transition from theory to practice. Circ J. (2010) 74:213–20. 10.1253/circj.CJ-09-0706 20065609

[33] Wu G Zhang J Zhao Q Zhuang W Ding J Zhang C. Molecularly engineered macrophage-derived exosomes with inflammation tropism and intrinsic heme biosynthesis for atherosclerosis treatment. Angew Chem Int Ed Engl. (2020) 59:4068–74. 10.1002/anie.201913700 31854064

[34] Liu Y Li C Wu H Xie X Sun Y Dai M. Paeonol attenuated inflammatory response of endothelial cells via stimulating monocytes-derived exosomal microRNA-223. Front Pharmacol. (2018) 9:1105. 10.3389/fphar.2018.01105 30515094 PMC6256086

[35] Shi C Ulke-Lemée A Deng J Batulan Z O'Brien ER. Characterization of heat shock protein 27 in extracellular vesicles: a potential anti-inflammatory therapy. FASEB J. (2019) 33:1617–30. 10.1096/fj.201800987R 30188755

[36] Puhm F Afonyushkin T Resch U Obermayer G Rohde M Penz T. Mitochondria are a subset of extracellular vesicles released by activated monocytes and induce type I IFN and TNF responses in endothelial cells. Circ Res. (2019) 125:43–52. 10.1161/CIRCRESAHA.118.314601 31219742

[37] Zhu J Liu B Wang Z Wang D Ni H Zhang L. Exosomes from nicotine-stimulated macrophages accelerate atherosclerosis through miR-21-3p/PTEN-mediated VSMC migration and proliferation. Theranostics. (2019) 9:6901–19. 10.7150/thno.37357 31660076 PMC6815950

[38] Yang S Xia YP Luo XY Chen SL Li BW Ye ZM. Exosomal CagA derived from *Helicobacter pylori*-infected gastric epithelial cells induces macrophage foam cell formation and promotes atherosclerosis. J Mol Cell Cardiol. (2019) 135:40–51. 10.1016/j.yjmcc.2019.07.011 31352044

[39] Minghua W Zhijian G Chahua H Qiang L Minxuan X Luqiao W. Plasma exosomes induced by remote ischaemic preconditioning attenuate myocardial ischaemia/reperfusion injury by transferring miR-24. Cell Death Dis. (2018) 9:320. 10.1038/s41419-018-0274-x 29476052 PMC5833738

[40] Gao XF Lu S Ge Z Zuo GF Wang ZM Wang F. Relationship between high platelet reactivity on clopidogrel and long-term clinical outcomes after drug-eluting stents implantation (PAINT-DES): a prospective, propensity score-matched cohort study. BMC Cardiovasc Disord. (2018) 18:103. 10.1186/s12872-018-0841-1 29793432 PMC5968524

[41] Wang Z Zhu H Shi H Zhao H Gao R Weng X. Exosomes derived from M1 macrophages aggravate neointimal hyperplasia following carotid artery injuries in mice through miR-222/CDKN1B/CDKN1C pathway. Cell Death Dis. (2019) 10:422. 10.1038/s41419-019-1667-1 31142732 PMC6541659

[42] Wang D Gao B Yue J Liu F Liu Y Fu W. Exosomes from mesenchymal stem cells expressing miR-125b inhibit neointimal hyperplasia via myosin IE. J Cell Mol Med. (2019) 23:1528–40. 10.1111/jcmm.14060 30484954 PMC6349157

[43] Hu H Wang B Jiang C Li R Zhao J. Endothelial progenitor cell-derived exosomes facilitate vascular endothelial cell repair through shuttling miR-21-5p to modulate Thrombospondin-1 expression. Clin Sci (Lond). (2019) 133:1629–44. 10.1042/CS20190188 31315970

[44] Bunggulawa EJ Wang W Yin T Wang N Durkan C Wang Y. Recent advancements in the use of exosomes as drug delivery systems. J Nanobiotechnol. (2018) 16:81. 10.1186/s12951-018-0403-9 30326899 PMC6190562

[45] Chistiakov DA Orekhov AN Bobryshev YV. Cardiac extracellular vesicles in normal and infarcted heart. Int J Mol Sci. (2016) 17:63. 10.3390/ijms17010063 26742038 PMC4730308

[46] Piffoux M Nicolás-Boluda A Mulens-Arias V Richard S Rahmi G Gazeau F. Extracellular vesicles for personalized medicine: The input of physically triggered production, loading and theranostic properties. Adv Drug Deliv Rev. (2019) 138:247–58. 10.1016/j.addr.2018.12.009 30553953

[47] Caplan AI Dennis JE. Mesenchymal stem cells as trophic mediators. J Cell Biochem. (2006) 98:1076–84. 10.1002/jcb.20886 16619257

[48] Yeo RW Lai RC Zhang B Tan SS Yin Y Teh BJ. Mesenchymal stem cell: an efficient mass producer of exosomes for drug delivery. Adv Drug Deliv Rev. (2013) 65:336–41. 10.1016/j.addr.2012.07.001 22780955

[49] Richter M Vader P Fuhrmann G. Approaches to surface engineering of extracellular vesicles. Adv Drug Deliv Rev. (2021) 173:416–26. 10.1016/j.addr.2021.03.020 33831479

[50] Kim MS Haney MJ Zhao Y Mahajan V Deygen I Klyachko NL. Development of exosome-encapsulated paclitaxel to overcome MDR in cancer cells. Nanomed Nanotechnol Biol Med. (2016) 12:655–64. 10.1016/j.nano.2015.10.012 26586551 PMC4809755

[51] Haney MJ Klyachko NL Zhao Y Gupta R Plotnikova EG He Z. Exosomes as drug delivery vehicles for Parkinson's disease therapy. J Control Release. (2015) 207:18–30. 10.1016/j.jconrel.2015.03.033 25836593 PMC4430381

[52] Elsharkasy OM Nordin JZ Hagey DW de Jong OG Schiffelers RM Andaloussi SE. Extracellular vesicles as drug delivery systems: Why and how? Adv Drug Deliv Rev. (2020) 159:332–43. 10.1016/j.addr.2020.04.004 32305351

[53] Kooijmans SAA Stremersch S Braeckmans K de Smedt SC Hendrix A Wood MJA. Electroporation-induced siRNA precipitation obscures the efficiency of siRNA loading into extracellular vesicles. J Control Release. (2013) 172:229–38. 10.1016/j.jconrel.2013.08.014 23994516

[54] Sutaria DS Jiang J Elgamal OA Pomeroy SM Badawi M Zhu X. Low active loading of cargo into engineered extracellular vesicles results in inefficient miRNA mimic delivery. J Extracell Vesicles. (2017) 6:1333882. 10.1080/20013078.2017.1333882 28717424 PMC5505005

[55] Banaszynski LA Liu CW Wandless TJ. Characterization of the FKBP.rapamycin.FRB ternary complex. J Am Chem Soc. (2005) 127:4715–21. 10.1021/ja043277y 15796538

[56] Yim N Ryu SW Choi K Lee KR Lee S Choi H. Exosome engineering for efficient intracellular delivery of soluble proteins using optically reversible protein-protein interaction module. Nat Commun. (2016) 7:12277. 10.1038/ncomms12277 27447450 PMC4961865

[57] Armstrong JPK Stevens MM. Strategic design of extracellular vesicle drug delivery systems. Adv Drug Deliv Rev. (2018) 130:12–6. 10.1016/j.addr.2018.06.017 29959959 PMC6606438

[58] Shen B Wu N Yang JM Gould SJ. Protein targeting to exosomes/microvesicles by plasma membrane anchors. J Biol Chem. (2011) 286:14383–95. 10.1074/jbc.M110.208660 21300796 PMC3077638

[59] Hong HY Lee HY Kwak W Yoo J Na MH So IS . Phage display selection of peptides that home toatherosclerotic plaques: IL-4 receptor as a candidate target in atherosclerosis. J Cell Mol Med. (2008) 12:2003–14. 10.1111/j.1582-4934.2008.00189.x 19012727 PMC4506166

[60] Lee GY Kim JH Oh GT Lee BH Kwon IC Kim IS. Molecular targeting of atherosclerotic plaques by a stabilin-2-specific peptide ligand. J Control Release. (2011) 155:211–7. 10.1016/j.jconrel.2011.07.010 21781994

[61] Won YW McGinn AN Lee M Bull DA Kim SW. Targeted gene delivery to ischemic myocardium by homing peptide-guided polymeric carrier. Mol Pharm. (2013) 10:378–85. 10.1021/mp300500y 23214982 PMC3542830

[62] Wang X Chen Y Zhao Z Meng Q Yu Y Sun J. Engineered exosomes with ischemic myocardium-targeting peptide for targeted therapy in myocardial infarction. J Am Heart Assoc. (2018) 7:e008737. 10.1161/JAHA.118.008737 30371236 PMC6201471

[63] Vandergriff A Huang K Shen D Hu S Hensley MT Caranasos TG. Targeting regenerative exosomes to myocardial infarction using cardiac homing peptide. Theranostics. (2018) 8:1869–78. 10.7150/thno.20524 29556361 PMC5858505

[64] Lv K Li Q Zhang L Wang Y Zhong Z Zhao J. Incorporation of small extracellular vesicles in sodium alginate hydrogel as a novel therapeutic strategy for myocardial infarction. Theranostics. (2019) 9:7403–16. 10.7150/thno.32637 31695776 PMC6831299

[65] Huang K Ozpinar EW Su T Tang J Shen D Qiao L. An off-the-shelf artificial cardiac patch improves cardiac repair after myocardial infarction in rats and pigs. Sci Transl Med. (2020) 12:eaat9683. 10.1126/scitranslmed.aat9683 32269164 PMC7293901

[66] Liu H Liu S Qiu X Yang X Bao L Pu F. Donor MSCs release apoptotic bodies to improve myocardial infarction via autophagy regulation in recipient cells. Autophagy. (2020) 16:2140–55. 10.1080/15548627.2020.1717128 31959090 PMC7751634

[67] Korin N Gounis MJ Wakhloo AK Ingber DE. Targeted drug delivery to flow-obstructed blood vessels using mechanically activated nanotherapeutics. JAMA Neurol. (2015) 72:119–22. 10.1001/jamaneurol.2014.2886 25365638

[68] Miyazaki Y Nomura S Miyake T Kagawa H Kitada C Taniguchi H. High shear stress can initiate both platelet aggregation and shedding of procoagulant containing microparticles. Blood. (1996) 88:3456–64. 10.1182/blood.V88.9.3456.bloodjournal8893456 8896411

[69] Staykova M Holmes DP Read C Stone HA. Mechanics of surface area regulation in cells examined with confined lipid membranes. Proc Natl Acad Sci U S A. (2011) 108:9084–8. 10.1073/pnas.1102358108 21562210 PMC3107321

[70] Savina A Fader CM Damiani MT Colombo MI. Rab11 promotes docking and fusion of multivesicular bodies in a calcium-dependent manner. Traffic (Copenhagen, Denmark). (2005) 6:131–43. 10.1111/j.1600-0854.2004.00257.x 15634213

[71] Gatzoulis MA Alonso-Gonzalez R Beghetti M. Pulmonary arterial hypertension in paediatric and adult patients with congenital heart disease. Eur Respir Rev. (2009) 18:154–61. 10.1183/09059180.00003309 20956136

[72] Chen YW Chen YC Wang JS. Absolute hypoxic exercise training enhances *in vitro* thrombin generation by increasing procoagulant platelet-derived microparticles under high shear stress in sedentary men. Clin Sci (Lond). (2013) 124:639–49. 10.1042/CS20120540 23252666

